# Morphological and molecular characterization of bacterial endophytes from *Centella asiatica* leaves

**DOI:** 10.1186/s43141-022-00456-8

**Published:** 2022-12-28

**Authors:** Siphiwe G. Mahlangu, Siew L. Tai

**Affiliations:** 1 Department of Chemical Engineering, Centre for Bioprocess Engineering Research, University of Cape Town, Private Bag X3, Rondebosch, Cape Town, 7701 South Africa; 2 Department of Chemical Engineering, Stellenbosch University, Private Bag X1, Matieland, 7602 South Africa

**Keywords:** *Centella asiatica*, Endophytic bacteria, 16S rRNA sequencing, Scanning electron microscopy, Phylogenetic analysis

## Abstract

**Background:**

Endophytes are a rich source of novel, distinct, and applicable compounds of interest in agricultural, medical, cosmetic, and pharmaceutical industries. In this respect, they have been attracting growing interest in the past few years. Endophytes are defined as microorganisms such as bacteria and fungi which have a mutualistic relationship with their host plants without causing any harm to their host. In this study, we isolated and identified bacterial endophytes from *Centella asiatica* collected in Western Cape, South Africa.

**Results:**

Twenty bacterial endophytes were isolated from *Centella asiatica* and characterized by using morphological and molecular techniques. Based on molecular traits, the isolates were identified as *Pseudomonas* sp. strain SGM1, *Pseudomonas* sp. strain SGM2, *Pseudomonas* sp. strain SGM3, *Pseudomonas* sp. strain SGM4, *Pseudomonas* sp. strain SGM5, *Pseudomonas* sp. strain SGM6, *Pseudomonas* sp. strain SGM7, *Novosphingobium* sp. strain SGM8, *Pseudomonas* sp. strain SGM9, *Pseudomonas* sp. strain SGM10, *Chryseobacterium* sp. strain SGM11, *Enterobacter* sp. strain SGM12, *Enterobacter* sp. strain SGM13, *Pseudomonas* sp. strain SGM14, *Enterobacter* sp. strain SGM15, *Enterobacter* sp. strain SGM16, *Agrobacterium* sp. strain SGM17, *Pantoea* sp. strain SGM18, *Paraburkholderia* sp. strain SGM19, and *Pseudomonas* sp. strain SGM20. *Pseudomonas* genus was dominant with eleven isolates. Morphological trait results showed that all isolates were gram-negative rod-shaped bacteria.

**Conclusion:**

According to our understanding, this study revealed the first twenty endophytic bacteria isolated from *Centella asiatica* growing in the Western Cape Province, South Africa. Data obtained in the current study will increase the knowledge of the already existing microbial diversity associated with *Centella asiatica*. Further work is needed to evaluate the antioxidant and antibacterial activities in vitro and assess the growth and medicinal compounds of the identified endophytic bacteria in a laboratory scale bioreactors.

## Background

*Centella asiatica* (L.) Urban (Apiaceae) is one of the most important high-value perennial herb that grows in temperate and tropical wetlands [1]. It is commonly known as Pennywort or *gotu kola* and normally grows in tropical wetlands and is native to humid and warmer regions of the hemisphere. It is distributed in the subtropical and tropical zones in India, minor parts of Venezuela, Madagascar, China, Mexico, South Africa, South-East America, and South-East Asian countries [2, 3].

Plant extracts have been used for medicinal purposes for thousands of years, and this includes treating various skin conditions such as eczema, leprosy, varicose ulcers, psoriasis, and lupus as well as treatment of female genitourinary tract diseases [4, 5]. Moreover, *C. asiatica* has been used as a brain tonic for improvement of memory and learning performance and incorporated into anti-inflammatory, antiaging, and antioxidant creams in the cosmetic industry. These medicinal properties are due to the presence of bioactive compounds as this herb is rich in triterpenoids, flavonoids, vitamins, tannins, polyphenol, and volatile oils. These chemical constituents are present in the whole plant but available in high content in the leaves [6–8].

In South Africa, *C. asiatica* is traditionally known as *Varkoortjies* or *Waternavel* (Afrikaans), *Udingu* (Xhosa), and *Umangobozane* or *Isgoba* (Zulu), and it is mostly found in the wet habitats of the southeastern Karoo, Western Cape, Eastern Cape, and KwaZulu-Natal regions of South Africa [1, 9]. *C. asiatica* is used as traditional herbal medicine by the indigenous people including traditional healers also known as *Sangomas* for the treatment of various ailments and diseases. This includes relieving ear pain in children by utilizing fresh leaves as ear plugs, treatment of rheumatoid arthritis, sharp internal body pains, wounds, syphilis, cancer, and acne [10–12]. It is also used to treat allergies, anemia, cholera, anxiety, constipation, bronchitis, and fever [13, 14]. Furthermore, *C. asiatica* has been proven to have a wide range of pharmacological activities due to the synthesized bioactive compounds known as centelloids and terpenoids. Centelloids consist of triterpenoids saponins as well as pentacyclic, whereas the terpenoids are made up of madecassic acids, centelloside, and asiaticoside to name a few. Some of the antimicrobial metabolites produced by *C. asiatica* are triterpenoids. Triterpenoids are regarded as phytoanticipins because they have antimicrobial properties that are utilized to eradicate pathogenic infections [15, 16]. Other than flavonoids and terpenoids, *C. asiatica* also has essential oil made up of trans-β-farnesene, bicyclogermacrene, myrcene, germacrene B and D, and *β*-caryophyllene with a broad spectrum of antibacterial activities against both gram-negative and gram-positive bacteria [11].

Endophytes are microbial species, either fungal or bacterial species that have a symbiotic relationship with plant species [17, 18]. Because they have a symbiotic relationship, some of the functions endophytes perform include the promotion of plant growth and development by solubilization of potassium and phosphate, production of growth hormones like cytokinin and auxin, improving the host plant overall health and growth by enhancing plant tolerance to different abiotic and biotic stresses, and protecting the plant from pathogenic species [19–21]. Moreover, endophytes can synthesize bioactive compounds that can be utilized as raw materials in various industries such as food, medicine, fragrance, and cosmetic industries [22–25]. Bacterial endophytes have been isolated from different plant parts (leaves, stems, roots, and fruits) of various medicinal plants. In addition, many reports have studied endophytes based on several microscopic visualizations and molecular techniques [26, 27]. Previous studies on endophytes from *C. asiatica* leaves have focused mostly on endophytic fungi, viz., *Aspergillus* sp., *Ceratobasidium* sp., *Fusarium* sp., *Phialemoniopsis* sp., *Colletotrichum* sp., *Glomerella* sp., *Guignardia* sp., *Nigrospora* sp., *Curvularia* sp., and *Colletotrichum* sp. [28–31]. Although the diversity of endophytic fungi and some endophytic bacteria have been isolated, identified, and characterized, reports on the occurrence of endophytic bacteria within *C. asiatica* are limited. In light of this, we report on the isolation of bacterial endophytes from the leaves of *Centella asiatica* collected in the Western Cape, South Africa region, which were identified on morphology using microscopic-based techniques and sequencing of 16S rRNA-based phylogeny.

## Methods

### Processing of plant samples

The fresh leaves of the medicinal plant *Centella asiatica* (L.) were harvested from their natural habitat in Constantia Heights, Cape Town (34.0058 32°S 18.43318°E), a part of the Western Cape Province, South Africa. The identification of the plant was done by Professor Cornelia Klak (Botanist), University of Cape Town Bolus Herbarium, with voucher number Stuart Hall 001(BOL). The isolation of bacterial endophytes was carried out at the Centre for Bioprocess Engineering Research (CeBER) laboratory of the Chemical Engineering Department, within 24 h of collection.

### Surface sterilization of leaves and isolation of endophytic bacterial isolates

Bacterial endophytes were isolated under aseptic conditions according to Mahlangu and Serepa-Dlamini [32]. The collected leaves were gently washed in running water to eradicate debris and dust. Samples were surface sterilized by 70% ethanol for 5 min, rinsed with sterile distilled water, and then treated with 2% sodium hypochlorite (NaClO) for 3 min. The sterilized leaves were finally rinsed with sterile distilled water, and the final wash was used as control and plated onto nutrient agar. The sterilized plant material was cut into 0.75 ± 0.25 cm pieces, crushed, and macerated with sterile PBS (phosphate-buffered saline, PH 7.4) for the isolation of bacterial endophytes. This was followed by streaking the homogenate onto nutrient agar plates. These plates were incubated together with control at 30 °C for 2–7 days with daily monitoring for bacterial growth and colonies. The different isolated colonies were selected based on visible morphological differences and subcultured on nutrient agar plates until pure cultures/colonies were obtained. Lastly, 30% glycerol stocks of the obtained pure bacterial cultures were prepared and stored at −80 °C for long-term storage and future use.

### Molecular identification and phylogenetic analysis of endophytic bacteria isolated from *Centella asiatica* leaves

For the DNA extraction, the Zymo Research Fungal/Bacterial kit (Zymo Research, USA) was used to isolate the DNA as per the manufacturer’s protocol. The 16S rRNA was amplified by polymerase chain reaction (PCR), using the primers 16S-27F: 5′-AGAGTTTGATCMTGGCTCAG-3′ and 16S-1492R: 5′-CGGTTACCTTGTTACGACTT-3′. The 16S rRNA sequence data were screened for chimeras using DECIPHER23 and subjected to Basic Local Alignment Search Tool (BLAST) analysis on National Centre for Biotechnology Information (NCBI) available at http://blast.ncbi.nlm.nih.gov. For phylogenetic analysis, BLAST was used to retrieve similar sequences from NCBI [17].

This was preceded by the alignment of the selected sequences with MUSCLE and the construction of phylogenetic trees using MEGA 11.0. The obtained phylogenetic trees were converted to Newick format, and the tree was further visualized using the Interactive Tree Of Life (iTOL) (https://itol.embl.de/) server [33].

### Microscopic visualization of endophytic bacterial isolates

The gram staining technique was performed to determine morphological characteristics (gram stain reaction, culture purity, and shape) of the isolates’ pure colonies. A compound bright-field microscope (OLYMPUS CH20BIMF200) at 100× magnification was used to view the gram stain slides [34].

### Scanning electron microscopy (SEM) analysis of endophytic bacterial isolates

Characterization by scanning electron microscopy (SEM) was performed to further determine the features of the endophytic isolates. The cultures were prepared using methods described by Kumar et al. [35] and with slight modifications. In brief, endophytic isolates were grown in 10 ml nutrient broth at 30 °C, shaking at 130 rpm for 48 h. The bacterial cultures were centrifuged for 10 min at 10,000 rpm, and the supernatant was discarded. Cells were then washed with sterile distilled water and fixed with 2.5% glutaraldehyde overnight. The samples were washed with distilled water, followed by dehydration with ethanol at concentrations of 30%, 50%, 70%, 90%, 95%, and 100% for 5 min each. Following dehydration, samples were centrifuged for 10 min at 10,000 rpm. Fixed and dehydrated pellets were filtered and glued onto aluminum stubs hexamethyldisilazane (HMDS) and mounted on stubs covered with carbon glue. The stubs were coated with carbon and evaluated by TESCAN MIRA SEM for viewing (Tescan-Orsay, Czech Republic).

## Results

### Molecular identification and phylogenetic analysis

The isolation of bacterial endophytes from the leaves of the medicinal plant *Centella asiatica* collected from the Western Cape region, South Africa, resulted in obtaining 20 bacterial strains. The obtained isolates were subjected to molecular identification by sequencing of the 16S rRNA gene amplification and compared with their closest match using the BLAST search tool program. The 16S rRNA sequences were deposited in GenBank, and isolates were designated new names and accession numbers as shown in Table 1.

**Table 1:** NCBI BLAST 16S rRNA gene sequences of bacterial endophytes isolated from *Centella asiatica* leaves

Assigned bacterial name	Assigned GenBank accession number	NCBI BLAST results		
		Closest related species with accession number	***e***-value	Identity similarity %
*Pseudomonas* sp. strain SGM1	MZ825291	*Pseudomonas* sp. CP025262.1, *Pseudomonas putida* MG836226.1	0.0	99.93
*Pseudomonas* sp. strain SGM2	MZ825292	*Pseudomonas moraviensis* MN752870.1, *Pseudomonas koreensis* MH011934.1 *Pseudomonas granadensis* MG269607.1	0.0	99.91
*Pseudomonas* sp. strain SGM3	MZ825293	*Pseudomonas* sp. DQ337600.1 *Pseudomonas putida* KJ569369.1	0.0	100
*Pseudomonas* sp. strain SGM4	MZ825294	*Pseudomonas moraviensis* MN752870.1 *Pseudomonas fluorescens* KT695833.1	0.0	99.85
*Pseudomonas* sp. strain SGM5	MZ825295	*Pseudomonas grimontii* KR054989.1	0.0	100
*Pseudomonas* sp. strain SGM6	MZ825296	*Pseudomonas* sp. JX067735.1 *Pseudomonas rhizosphaerae* CP009533.1	0.0	99.79
*Pseudomonas* sp. strain SGM7	MZ825297	*Pseudomonas chlororaphis* MT078671.1 *Pseudomonas koreensis* MN710458.1 *Pseudomonas fluorescens* MK719958.1	0.0	99.60
*Novosphingobium* sp. strain SGM8	MZ825298	*Novosphingobium clariflavum* NR_157981.1	0.0	99.34
*Pseudomonas* sp. strain SGM9	MZ825299	*Pseudomonas fluorescens* CP027561.1 *Pseudomonas allokribbensis* CP062252.1	0.0	99.07
*Pseudomonas* sp. strain SGM10	MZ825300	*Pseudomonas rhodesiae* CP054205.1	0.0	99.77
*Chryseobacterium* sp. strain SGM11	MZ825301	*Chryseobacterium* sp. AY468462.1 *Chryseobacterium scophthalmum* KC178594.1	0.0	99.17
*Enterobacter* sp. strain SGM12	MZ825302	*Enterobacter* sp. MH669343.1 *Enterobacter ludwigii* KC355280.1	0.0	100
*Enterobacter* sp. strain SGM13	MZ825303	*Enterobacter* sp. MH669343.1 *Enterobacter ludwigii* KC355280.1	0.0	99.93
*Pseudomonas* sp. strain SGM14	MZ825304	*Pseudomonas moraviensis* MN752870.1 *Pseudomonas granadensis* MG269607.1 *Pseudomonas fluorescens* KT695833.1	0.0	99.61
*Enterobacter* sp. strain SGM15	MZ825305	*Enterobacter* sp. MH669343.1 *Enterobacter ludwigii* KC355280.1	0.0	100
*Enterobacter* sp. strain SGM16	MZ825306	*Uncultured Erwinia* sp. MF457488.1 *Enterobacter cancerogenus* HE575594.1	0.0	99.64
*Agrobacterium* sp. strain SGM17	MZ825307	*Agrobacterium vitis* MT367798.1	0.0	97.67
*Pantoea* sp. strain SGM18	MZ825308	*Pantoea agglomerans* MT367719.1 *Pantoea brenneri* KX588583.1 *Pantoea conspicua* MF083088.1	0.0	99.64
*Paraburkholderia* sp. strain SGM19	MZ825309	*Paraburkholderia caledonica* MN595030.1	0.0	99.86
*Pseudomonas* sp. strain SGM20	MZ825310	*Pseudomonas coleopterorum* NR_137215.1	0.0	99.79

**Fig. 1:**
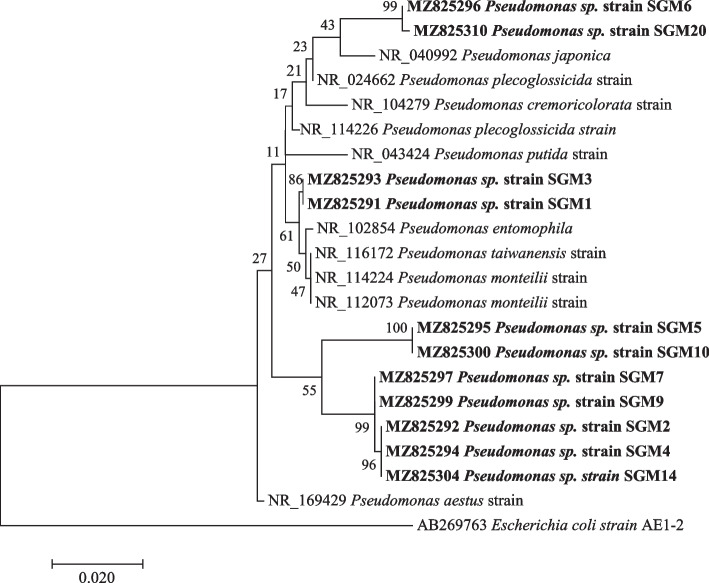
Maximum likelihood phylogenetic tree based on analysis of partial 16S rRNA nucleotide sequences of *Pseudomonas* sp. strain SGM1, *Pseudomonas* sp. strain SGM2, *Pseudomonas* sp. strain SGM3, *Pseudomonas* sp. strain SGM4, *Pseudomonas* sp. strain SGM5, *Pseudomonas* sp. strain SGM6, *Pseudomonas* sp. strain SGM7, *Pseudomonas* sp. strain SGM9, *Pseudomonas* sp. strain SGM10, *Pseudomonas* sp. strain SGM14, and *Pseudomonas* sp. strain SGM20 with reference sequences selected from the NCBI*. Escherichia coli strain* AE1-2 was used as an outgroup

**Fig. 2:**
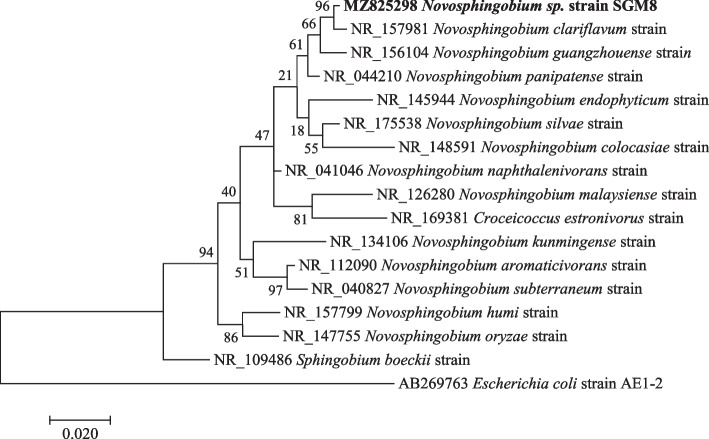
Maximum likelihood phylogenetic tree based on analysis of partial 16S rRNA nucleotide sequence of *Novosphingobium* sp. strain SGM8 with reference sequences selected from the NCBI*. Escherichia coli* strain AE1-2 was used as an outgroup

**Fig. 3:**
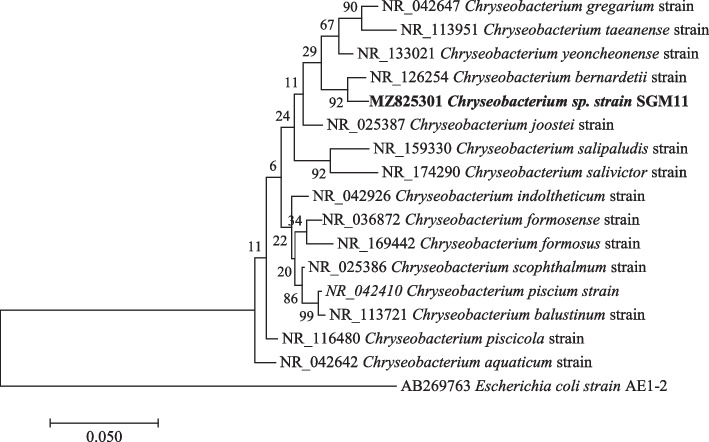
Maximum likelihood phylogenetic tree based on analysis of partial 16S rRNA nucleotide sequence of *Chryseobacterium* sp. strain SGM11 with reference sequences selected from the NCBI*. Escherichia coli strain* AE1-2 was used as an outgroup

**Fig. 4:**
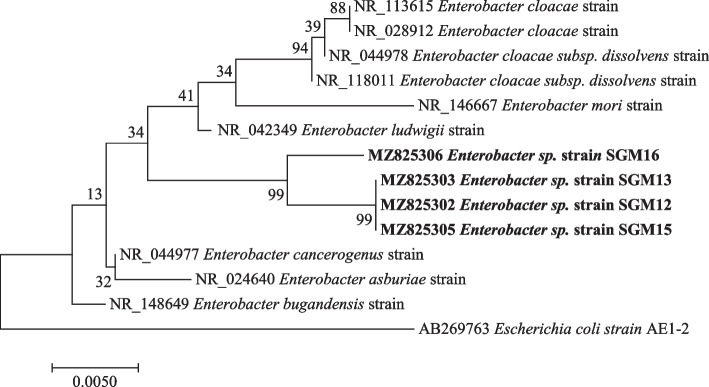
Maximum likelihood phylogenetic tree based on analysis of partial 16S rRNA nucleotide sequences of *Enterobacter* sp. strain SGM12, *Enterobacter* sp. strain SGM13, *Enterobacter* sp. strain SGM15, and *Enterobacter* sp. strain SGM16 with reference sequences selected from the NCBI*. Escherichia coli* strain AE1-2 was used as an outgroup

**Fig. 5:**
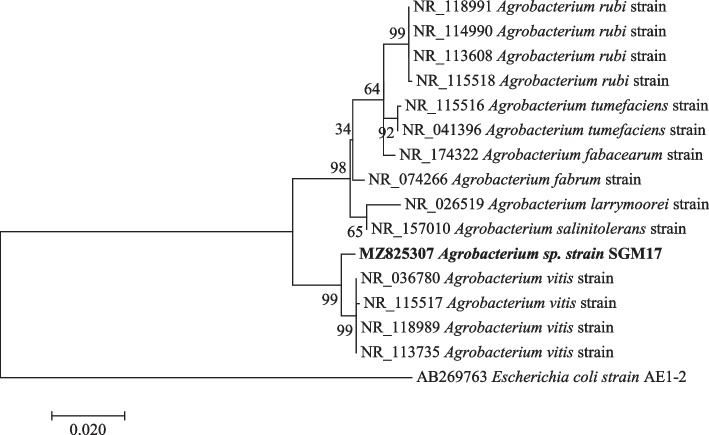
Maximum likelihood phylogenetic tree based on analysis of partial 16S rRNA nucleotide sequence of *Agrobacterium* sp. strain SGM17 with reference sequences selected from the NCBI*. Escherichia coli strain* AE1-2 was used as an outgroup

**Fig. 6:**
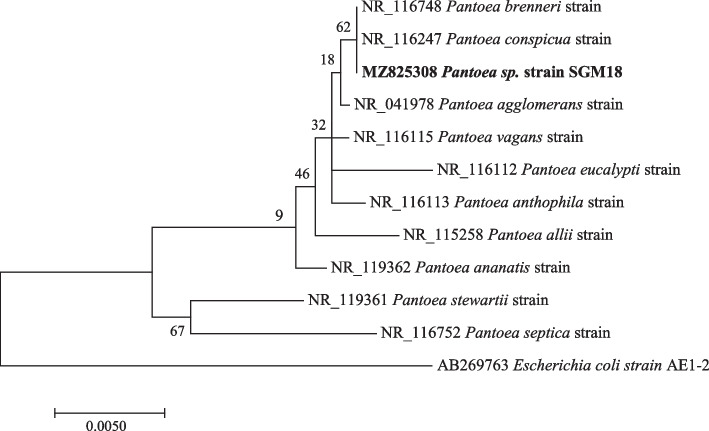
Maximum likelihood phylogenetic tree based on analysis of partial 16S rRNA nucleotide sequence of *Pantoea* sp. strain SGM18 with reference sequences selected from the NCBI. *Escherichia coli* strain AE1-2 was used as an outgroup

**Fig. 7:**
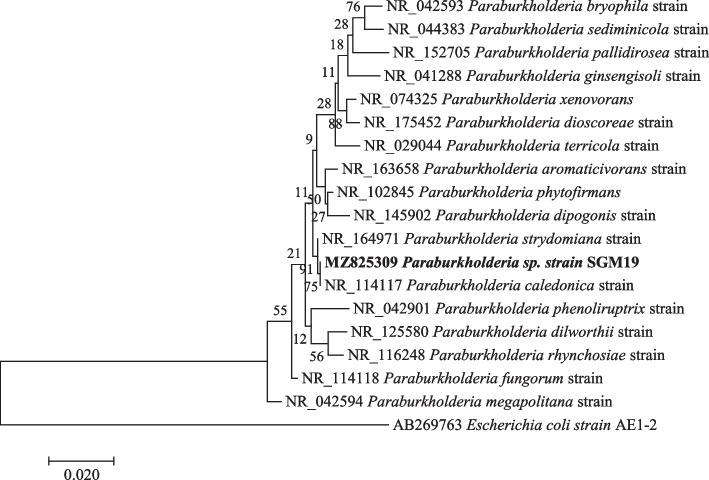
Maximum likelihood phylogenetic tree based on analysis of partial 16S rRNA nucleotide sequence of *Paraburkholderia* sp. strain SGM19 with reference sequences selected from the NCBI*. Escherichia coli* strain AE1-2 was used as an outgroup

**Fig. 8:**
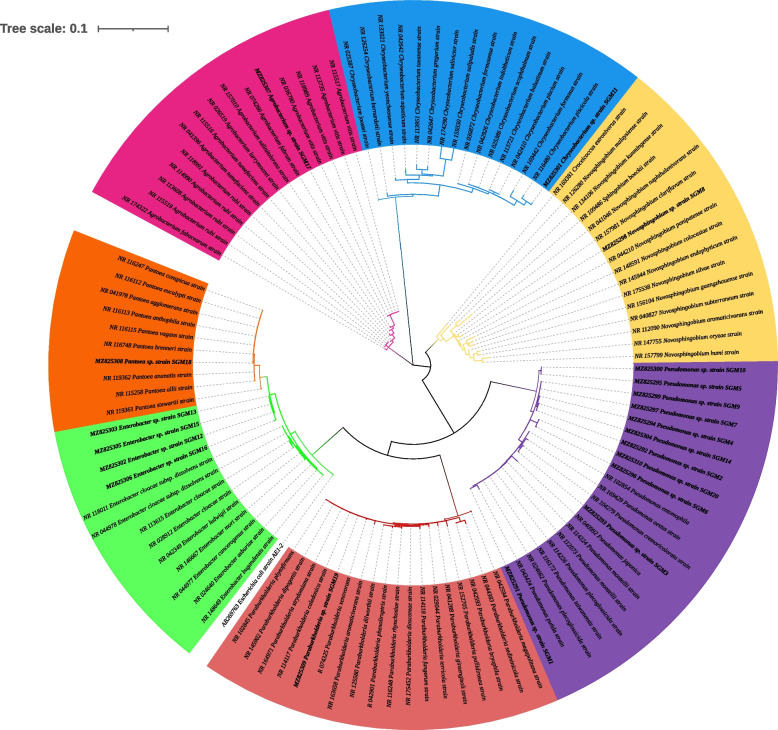
Maximum likelihood phylogenetic tree constructed using the 16S rRNA gene sequences showing the relationship of the 20 endophytic bacteria of this study with closely related species. The phylogenetic tree was generated using MEGA version 11.0 with bootstrap value above 70%, 1000 replications, and visualized with Interactive Trees Of Life (iTOL). The tree is colored to indicate the different genus’s; agrobacterium, pink; *Chryseobacterium*, blue; *Novosphingobium*; yellow, *Pseudomonas*; purple; *Paraburkholderia*, red; *Enterobacter*, green; and *Pantoea*, orange

Phylogenetic investigations were performed on all the strains with at least 99–100% nucleotide sequence similarity, with a 1000 bootstrap value using the maximum likelihood method. The sequences obtained in this study are represented by bold branch nodes (Figs. 1, 2, 3, 4, 5, 6, 7 and 8), whereas the other sequences are from the NCBI database and were used for comparing results.

### Morphological identification

**Fig. 9:**
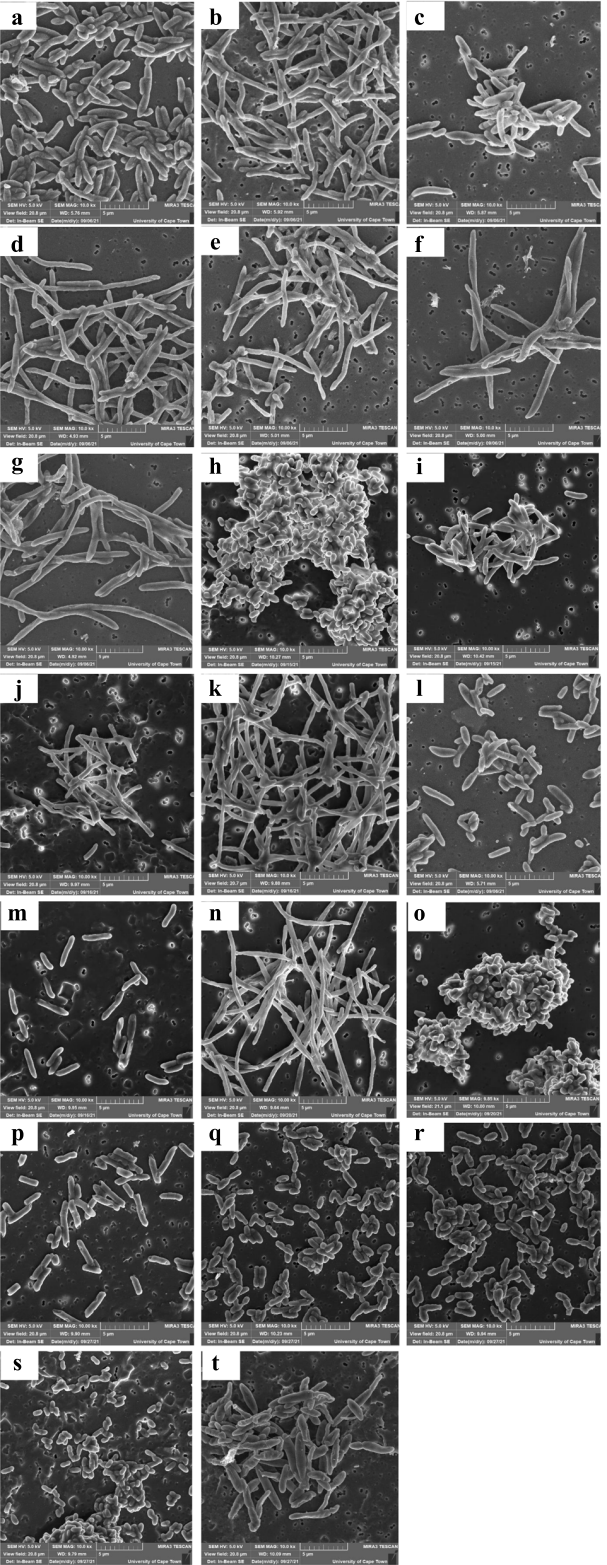
The scanning electron microscopy (SEM) images showing the evident morphological characteristics such as shape and size of the 20 endophytic isolates under study viewed at 10.0 k× magnification. **a** *Pseudomonas* sp. strain SGM1, **b** *Pseudomonas* sp. strain SGM2, **c** *Pseudomonas* sp. strain SGM3, **d** *Pseudomonas* sp. strain SGM4, **e** *Pseudomonas* sp. strain SGM5, **f** *Pseudomonas* sp. strain SGM6, **g** *Pseudomonas* sp. strain SGM7, **h** *Novosphingobium sp.* strain SGM8, **i** *Pseudomonas* sp. strain SGM9, **j** *Pseudomonas* sp. strain SGM10, **k** *Chryseobacterium* sp. strain SGM11, **l** *Enterobacter* sp. strain SGM12, **m** *Enterobacter* sp. strain SGM13, **n** *Pseudomonas* sp. strain SGM14, **o** *Enterobacter* sp. strain SGM15, ***p*** *Enterobacter* sp. strain SGM16, **q** *Agrobacterium* sp. strain SGM17, **r** *Pantoea* sp. strain SGM18, **s** *Paraburkholderia* sp. strain SGM19, and **t** Pseudomonas sp. strain SGM20

The morphology technique was used to identify the bacterial strains, and the Gram stain results indicated all the 20 endophytic bacteria as gram negative and rod shaped (Table 2). The scanning electron microscopy imaging was used for further verification of the bacterial shape, and uniformity was observed in the images as shown in Fig. 9 which indicated that the bacterial strains were pure cultures. Also, SEM imaging further confirmed the shape of the bacteria, and they were found to be rod shaped as indicated before by the gram straining technique.

**Table 2:** Morphological characteristics of bacterial endophytes isolated from *Centella asiatica* leaves

Bacterial sample code	Assigned isolate name	Phyla	Gram reaction	Cell shape
CA-BE1	*Pseudomonas* sp. strain SGM1	Proteobacteria	-ve	Rods
CA-BE2	*Pseudomonas* sp. strain SGM2	Proteobacteria	-ve	Rods
CA-BE3	*Pseudomonas* sp. strain SGM3	Proteobacteria	-ve	Rods
CA-BE4	*Pseudomonas* sp. strain SGM4	Proteobacteria	-ve	Rods
CA-BE5	*Pseudomonas* sp. strain SGM5	Proteobacteria	-ve	Rods
CA-BE6	*Pseudomonas* sp. strain SGM6	Proteobacteria	-ve	Rods
CA-BE7	*Pseudomonas* sp. strain SGM7	Proteobacteria	-ve	Rods
CA-BE8	*Novosphingobium* sp. strain SGM8	Proteobacteria	-ve	Rods
CA-BE9	*Pseudomonas* sp. strain SGM9	Proteobacteria	-ve	Rods
CA-BE10	*Pseudomonas* sp. strain SGM10	Proteobacteria	-ve	Rods
CA-BE11	*Chryseobacterium* sp. strain SGM11	Bacteroidetes	-ve	Rods
CA-BE12	*Enterobacter* sp. strain SGM12	Proteobacteria	-ve	Rods
CA-BE13	*Enterobacter* sp. strain SGM13	Proteobacteria	-ve	Rods
CA-BE14	*Pseudomonas* sp. strain SGM14	Proteobacteria	-ve	Rods
CA-BE15	*Enterobacter* sp. strain SGM15	Proteobacteria	-ve	Rods
CA-BE16	*Enterobacter* sp. strain SGM16	Proteobacteria	-ve	Rods
CA-BE17	*Agrobacterium* sp. strain SGM17	Proteobacteria	-ve	Rods
CA-BE18	*Pantoea* sp. strain SGM18	Proteobacteria	-ve	Rods
CA-BE19	*Paraburkholderia* sp. strain SGM19	Proteobacteria	-ve	Rods
CA-BE20	*Pseudomonas* sp. strain SGM20	Proteobacteria	-ve	Rods

*Gram reaction: *-ve*, gram negative

## Discussion

*Centella asiatica* harbors a rich taxonomic diversity of endophytes; however, many studies have focused on fungal endophytes, and very few have explored endophytic bacteria. In this study, twenty bacterial endophytes (Table 1) were isolated from the leaves of *C. asiatica* and identified as *Pseudomonas* sp. strain SGM1, *Pseudomonas* sp. strain SGM2, *Pseudomonas* sp. strain SGM3, *Pseudomonas* sp. strain SGM4, *Pseudomonas* sp. strain SGM5, *Pseudomonas* sp. strain SGM6, *Pseudomonas* sp. strain SGM7, *Novosphingobium* sp. strain SGM8, *Pseudomonas* sp. strain SGM9, *Pseudomonas* sp. strain SGM10, *Chryseobacterium* sp. strain SGM11, *Enterobacter* sp. strain SGM12, *Enterobacter* sp. strain SGM13, *Pseudomonas* sp. strain SGM14, *Enterobacter* sp. strain SGM15, *Enterobacter* sp. strain SGM16, *Agrobacterium* sp. strain SGM17, *Pantoea* sp. strain SGM18, *Paraburkholderia* sp. strain SGM19, and *Pseudomonas* sp. strain SGM20. These isolates were classified into two phyla: Bacteroidetes and Proteobacteria (Table 2).

*Pseudomonas* and *Enterobacter* were dominant species with eleven and four endophytes respectively. Some bacterial endophytic strains of *Xanthomonas axonopodis*, *Pseudomonas fulva*, *Providencia vermicola*, *Erwinia* sp., *Pantoea agglomerans*, *Methylobacterium radiotolerans*, and *Bacillus gibsonii* were isolated from *C. asiatica* leaf petioles and stems and reported in 2012 [36]. Ernawati et al. [37] identified six genera: *Gordonia*, *Actinoplanes*, *Couchioplanes*, *Verrucosispora*, *Streptomyces*, and *Micromonospora* of *C. asiatica* from Indonesia. Thirty-one bacterial strains classified into the genera *Bacillus* sp., *Cohnella* sp., *Acinetobacter* sp., *Paenibacillus* sp., *Microbacterium* sp., *Achromobacter* sp., *Lysinibacillus* sp., *Pseudomonas* sp., *Pantoea* sp., *Klebsiella* sp., and *Delftia* sp. were reported earlier from surface-disinfected *C. asiatica* leaves, and they showed the capability to reduce the disease occurrence and growth rate of the hemibiotrophic fungus *Colletotrichum higginsianum* [38].

Seemingly, the most dominant genera of bacterial endophytes from *C. asiatica* are *Pseudomonas*, *Pantoea*, and *Bacillus*. Besides *Pseudomonas*, *Pantoea*, and *Bacillus*, other common fungal endophytes such as *Fusarium* and *Colletotrichum* have also been identified from *C. asiatica* [28, 30, 31]. Martín-García et al. (2011) noted that the diversity of the endophytic community of bacterial endophytes relies on several factors such as the geographical location, plant age, species, and cultivation conditions [39]. For example, nine bacterial endophytes were isolated from aerial parts of *C. asiatica* harvested in Malaysia [36]. In another study, three bacterial endophytes were isolated from the leaves of subtropical forest-cultivated *C. asiatica* in Meghalaya, India [40]. This result is in agreement with the statement above made by Martín-García et al. (2011) and Liu et al. (2017) for further stating that growth factors such as soil pH, annual temperature, organic matter, annual rainfall, and phosphate availability could result in variation in the distribution and composition of bacterial endophytes [41]. In other studies, endophytes isolated (mostly fungi) from *C. asiatica* were tested in vitro for their antioxidant, antimicrobial, and plant growth-promoting activities, and these studies proved these isolates to be good candidates with pharmaceutical importance and for application as biocontrol and biofertilizer agents [42–44].

Phylogenetic analysis showed that strain *Pseudomonas* sp. strain SGM20 had a biphyletic cluster with *Pseudomonas* sp. strain SGM6 (Fig. 1). In addition, other *Pseudomonas* sp. strains under study were closely related to each other (Fig. 1). As shown in Figs. 2 and 3, a sister relation was observed between *Novosphingobium* sp. strain SGM8 and *Novosphingobium clariflavum* strain supported by a 96% bootstrap value, whereas *Chryseobacterium* sp. strain SGM11 is closest to *Chryseobacterium bernardetii* strain supported by a 92% bootstrap value. Phylogenetic analysis further revealed that *Enterobacter* sp. strain SGM12, *Enterobacter* sp. strain SGM13, *Enterobacter* sp. strain SGM15, *Enterobacter* sp. strain SGM16 (Fig. 4), and *Agrobacterium* sp. strain SGM17 (Fig. 5) were clustered and formed a separate lineage. Therefore, the phylogenetic positioning of *Enterobacter* sp. strain SGM12, *Enterobacter* sp. strain SGM13, *Enterobacter* sp. strain SGM15, *Enterobacter* sp. strain SGM16, and *Agrobacterium* sp. strain SGM17 is an indication that these species are novel members of the *Enterobacter* and *Agrobacterium* genus.

*Pantoea* species revealed *Pantoea* sp. strain SGM18 had a polyphyletic relationship with *Pantoea brenneri* strain and *Pantoea conspicua* strain (Fig. 6), while *Paraburkholderia* sp. *strain* SGM19 was closely related to *Paraburkholderia caledonica* strain supported by a 75% bootstrap value (Fig. 7). The isolated endophytic bacteria were identified at the genus level using sequencing of 16S rRNA. However, the evolutionary and phylogenetic relationships between bacterial endophytes from the genera, *Pseudomonas*, *Enterobacter*, and *Pantoea* species, and closely related endophytic strains were not resolved as polyphyletic relationship was observed from the results obtained. Therefore, for phylogenetic delineation and species description, it is suggested to identify and further phylogenetically analyze the bacterial endophytes from the three genera using the multilocus sequencing analysis (MLSA) [27, 45].

Based on the morphological analysis, unique morphological characteristics such as colony size, shape, color, and margins were observed for each isolate (data not shown). According to our results, all isolated endophytic bacteria were gram-negative, rod-shaped bacteria. The scanning electron microscopy results further showed distinct characteristics in terms of the shape and size of the pure cultures from small to long rod-shaped bacteria (Fig. 9). Although in some studies the quantity of gram negative is equal to that of the gram positive, it has been reported that gram-negative bacterial endophytes are more likely to be abundant than gram-positive bacteria [46]. Therefore, this supports the obtained results in our study.

## Conclusion

Many researchers have isolated, identified, and reported on endophytes from *C. asiatica*, but these endophytes are mostly fungal, and very minimal research has been done on bacterial endophytes. This is also the first reported work on bacterial endophytes associated with *C. asiatica* isolated in South Africa. The different isolated colony diversity compared to those isolated from subtropical/tropical countries compared to a more Mediterranean climate that the Cape Town, Western Cape region, experiences confirms that geographical differences play a large role for endophyte colonization in plants. Therefore, more research work is necessary to understand how the diversity of the local bacterial endophytes establishes themselves and how these affect the application of these medicinal plants from an application standpoint.

Although *C. asiatica* is traditionally used as a medicinal plant within skin healthcare, there is little correlation to link these properties to endophytes, despite the numerous studies done to identify isolates. Further investigations in growing endophytes out of its host system and analysis into antioxidant and antimicrobial in vitro assays would therefore enable a systematic and formal approach in the discovery of novel biotherapeutic remedies and subsequently scaling up for large-scale (mass production) trials in bioreactors for pharmaceutical bioprospection.

## Data Availability

Supplementary data generated in this study can be download at https://figshare.com/s/1d23faa8c8784d2db9ef.

## Abbreviations

NA: Nutrient agar
NB: Nutrient broth
PBS: Phosphate-buffered saline
BLAST: Basic Local Alignment Search Tool
NCBI: National Centre for Biotechnology Information
SEM: Scanning electron microscope
HMDS: Hexamethyldisilazane
iTOL: Interactive Tree Of Life
